# A Systematic Review on the Effect of Saffron Extract on Lipid Profile in Hyperlipidaemic Experimental Animal Models

**DOI:** 10.21315/mjms2022.29.4.3

**Published:** 2022-08-29

**Authors:** Iman Nabilah Abd Rahim, Noor Alicezah Mohd Kasim, Mohamad Rodi Isa, Hapizah Nawawi

**Affiliations:** 1 Institute of Pathology, Laboratory and Forensic Medicine (I-PperForM), Universiti Teknologi MARA, Selangor, Malaysia; 2 Department of Pathology, Faculty of Medicine, Universiti Teknologi MARA, Selangor, Malaysia; 3 Department of Population Health and Preventive Medicine, Faculty of Medicine, Universiti Teknologi MARA, Selangor, Malaysia

**Keywords:** saffron, lipid profile, atherosclerosis, hyperlipidaemia, animal models

## Abstract

Saffron is widely used in traditional medicine to treat various medical disorders, including hyperlipidaemia. This study aims to systematically review the effects of saffron extract (SE) on lipid profile in in vivo studies. A strategic literature search was done following Preferred Reporting Items for Systematic Reviews and Meta-Analyses (PRISMA) guidelines. The Scopus, PubMed (MEDLINE) and Web of Science databases and hand-searching methods were utilised to identify studies published up to January 2020 that reported the effects of SE on lipid profile in a hyperlipidaemic experimental animal model. A total of six articles met the inclusion criteria. The methods of extraction were aqueous (*n* = 4), ethanolic (*n* = 1) and hydroalcoholic (*n* = 1) extracts. Five doses of SE ranging from 10 mg/kg to 100 mg/kg were administered to rats and hamsters, with a duration ranging from 10 days to 8 weeks. SE at doses of 40 mg/kg/day and 80 mg/kg/day significantly decreased the levels of total cholesterol (21.4%–35.4%), low-density lipoproteins (38.7%–50.0%) and triglycerides (TGs) (29.1%–45.0%) and markedly increased the level of high-density lipoproteins (36.6%–65%) in the treated group compared to the untreated group with a minimum 3-week intervention duration (*P* < 0.05). This systematic review demonstrated that SE exhibits hypolipidaemic effects compared to a placebo. SE has almost the same ability to reduce cholesterol levels as the standard therapy.

## Introduction

Dyslipidaemia is an abnormal condition characterised by an imbalance between the levels of low-density lipoprotein (LDL) and high-density lipoprotein (HDL) cholesterol. High LDL levels may lead to plaque formation within the arteries, whereas HDL decreases the risk. Thus, in dyslipidaemia, where LDL levels are high and HDL levels are low, there is a high likelihood of atherosclerotic cardiovascular disease. Along with the imbalance in cholesterol levels, hypertriglyceridaemia, which is an increased level of triglycerides (TGs) in the body, is another type of dyslipidaemia. Additionally, mixed hyperlipidaemia occurs when there is an imbalance in both the cholesterol and TG levels (1). Hyperlipidaemia can lead to coronary heart disease (CHD), which increases the morbidity and mortality in developing and developed countries (2). Hyperlipidaemia is a major risk for atherosclerosis and its related complications as well as a well-documented risk factor for cardiovascular diseases (3). According to the World Health Organization (WHO) (4), one-third of ischaemic heart disease cases worldwide are caused by high cholesterol levels. Overall, it is estimated that 4.5% of total deaths globally, accounting for 2.6 million cases and 2% of total disability-adjusted life years, which is about 29.7 million cases, are a result of elevated cholesterol levels (4).

In light of the severity of atherosclerosis and its related complications (e.g. CHD), it is crucial to examine the current treatment for hyperlipidaemia. Regarding pharmacological management, the treatment options are not free of adverse effects. As the mainstay of treatment, statins are widely used in managing this non-communicable disease. However, these drugs have some shortcomings, including resistance to treatment, intolerance due to adverse reactions and lack of patient compliance to the medication. These can cause a poor outcome, leading to the need for adjunctive therapies, such as niacin, bile acid sequestrants, fibric acid and ezetimibe, all of which have their own side effects (1). Due to these side effects and contraindications for long-term usage, it is vital that some types of natural hypolipidaemic drugs are utilised to prevent and manage the disease and its complications (5).

Saffron is part of the *Crocus sativus* L. flower, taken from the thread-like part of the flower, called the stigma, when it is dried. For the past 2,500 years, people have been using saffron as a medicine due to its numerous benefits. Apart from its utilisation in traditional medicine, saffron is also used as a spice in food and in colouring agents. It is the highest-priced spice globally, which is attributable to the method of growing and harvesting this flower (6). In traditional medicine, saffron has been used in cases of depression, cardiovascular diseases, menstruation disorders, asthma, insomnia, digestive ailments and some other diseases. The beneficial effects seen in these cases may be due to some of its components, namely, safranal, crocetin and crocin (7).

In the case of hyperlipidaemia, evidence suggests that cholesterol levels were lowered and maintained at healthy levels with the consumption of saffron (8). Both in vitro and in vivo studies reported that saffron extract (SE) has beneficial effects on lipid profile. For example, the results of an animal study showed that saffron improves malondialdehyde and lipid profile in diabetic rats (7). Other animal studies reported that utilisation of saffron was associated with a 50% reduction in cholesterol levels (8). In addition, as an antioxidant and anti-inflammatory substance, saffron is also useful in maintaining a healthy cardiovascular system (8).

To date, few systematic reviews have been conducted on the effect of saffron on either preventing or treating hyperlipidaemia. Further, the most efficient dose and minimum duration of treatment are not known. Thus, this study aims to review the effects of SE on lipid profile in in vivo studies, focusing on the following question: In hyperlipidaemic experimental animal models, does SE consumption result in anti-hyperlipidaemic or hypolipidaemic effects when compared with a placebo or standard therapy?

## Methods

This systematic review has been registered with the International Prospective Register of Systematic Reviews (PROSPERO) (identifier CRD42020159520). This review follows the standards listed in the Preferred Reporting Items for Systematic Reviews and Meta-Analyses (PRISMA) checklist (9). A search of published studies was performed using online databases, including Scopus, MEDLINE via PubMed and Web of Science, as well as hand-searching methods.

### Inclusion and Exclusion Criteria

Articles were included if the following criteria were fulfilled: i) the study assessed the effects of saffron on lipid changes in in vivo studies; ii) only *Crocus sativus* L. (saffron) stigma was used, not other parts of the plant, such as the petals or roots; iii) it was an original full article; iv) studies published in English language due to a lack of translation resources and v) studies published up to January 2020. The exclusion criteria were as follows: i) it was a Review Article, Case Report or Letters to the Editor; ii) the study conducted clinical trials; iii) the study was presented as an abstract only; iv) the study assessed the effect of SE on unrelated blood or clinical parameters; v) the study investigated the effect of SE in combination with other plants or exercise and vi) the study did not clearly mention the type of SE used.

### Searching Strategy

A systematic review of the literature was conducted to investigate relevant studies related to the effects of SE on lipid profile in in vivo studies. The formulation of the research question was based on the population, intervention, comparison and outcome (PICO) strategy. The research question for this study was ‘In hyperlipidaemic experimental animal model, does saffron compared to placebo or no treatment/standard therapy demonstrate anti-hyperlipidaemic and hypolipidaemic effects in in vivo studies?’

In 2020, we searched MEDLINE [276], Web of Science [477] and Scopus [583] for studies reviewing the effects of SE on lipid profile in in vivo studies published in journals between January 2010 and January 2020. The key search terms were: saffron OR *crocus sativus* OR crocin AND lipid* OR cholesterol* OR triglyceride* OR hyperlipid$emi* OR atherosclerosis OR heart OR cardi* OR lipid profile OR dyslipid$emi*.

### Study Selection

All citations identified from the search were downloaded into EndNote X7 software. The citations were organised, and duplicates were identified and deleted. Two independent reviewers conducted the study selection. If there were any discrepancies in the selection process, a third person was called to confirm the selection of articles.

### Data Extraction

Articles published up to January 2020 were selected. First, based on the title and abstracts of the research papers alone, articles that did not meet the inclusion criteria were excluded. Then, we further excluded articles that did not match the inclusion criteria after an in-depth reading of the full-text research papers. For duplicate publications, only the latest version was included. The most updated version was included in the review in case of duplicate publications. The data of interest were extracted and the following data were documented from the studies: i) aim of the study; ii) a brief description of the subject; iii) type, dose and route of the extract used; iv) a brief description of the results and v) the conclusion of the study. To determine the potential anti-dyslipidaemic activity of SE in in vivo studies, several principal parameters were evaluated, including lipid profile measurement (TG, TC, LDL and HDL).

### Review Methods

All potential articles were identified and screened by two independent reviewers. Study selection and data extraction were performed following detailed discussion and any disagreements or uncertainties about the study inclusion was resolved.

### Data Analysis

Descriptive analyses of the characteristics of the studies and statistical methods used were performed. This is a descriptive review and all results are displayed as frequencies to determine how many studies reported positive and negative effects of SE on lipid profile in vivo. Table 1 summarises the effects of SE on lipid profile (TG, TC, HDL and LDL) in a hyperlipidaemic experimental animal model. The data analyses were performed using Microsoft Excel 2016 (Microsoft Office).

## Results

### Search Results or Study Selection

After searching, a total of 1,340 articles were found. An initial search of the electronic databases identified 1,339 articles and one article was identified through the hand-searching method, where the article was found on the reference list of one of the total articles. A total of 336 studies were excluded because they were not original articles and not written in English. After screening the titles and abstracts of the remaining articles, 946 articles were further removed because they were found to be unrelated to the objective; they were clinical trials, irrelevant or did not meet the inclusion criteria.

Of the remaining 58 articles, 31 were left after duplicates were removed. The full texts of one article could not be retrieved. Upon scrutiny of the available full texts, 25 articles were found to be unsatisfactory and discarded. Therefore, a total of six articles were included in this review.

**Figure 1:**
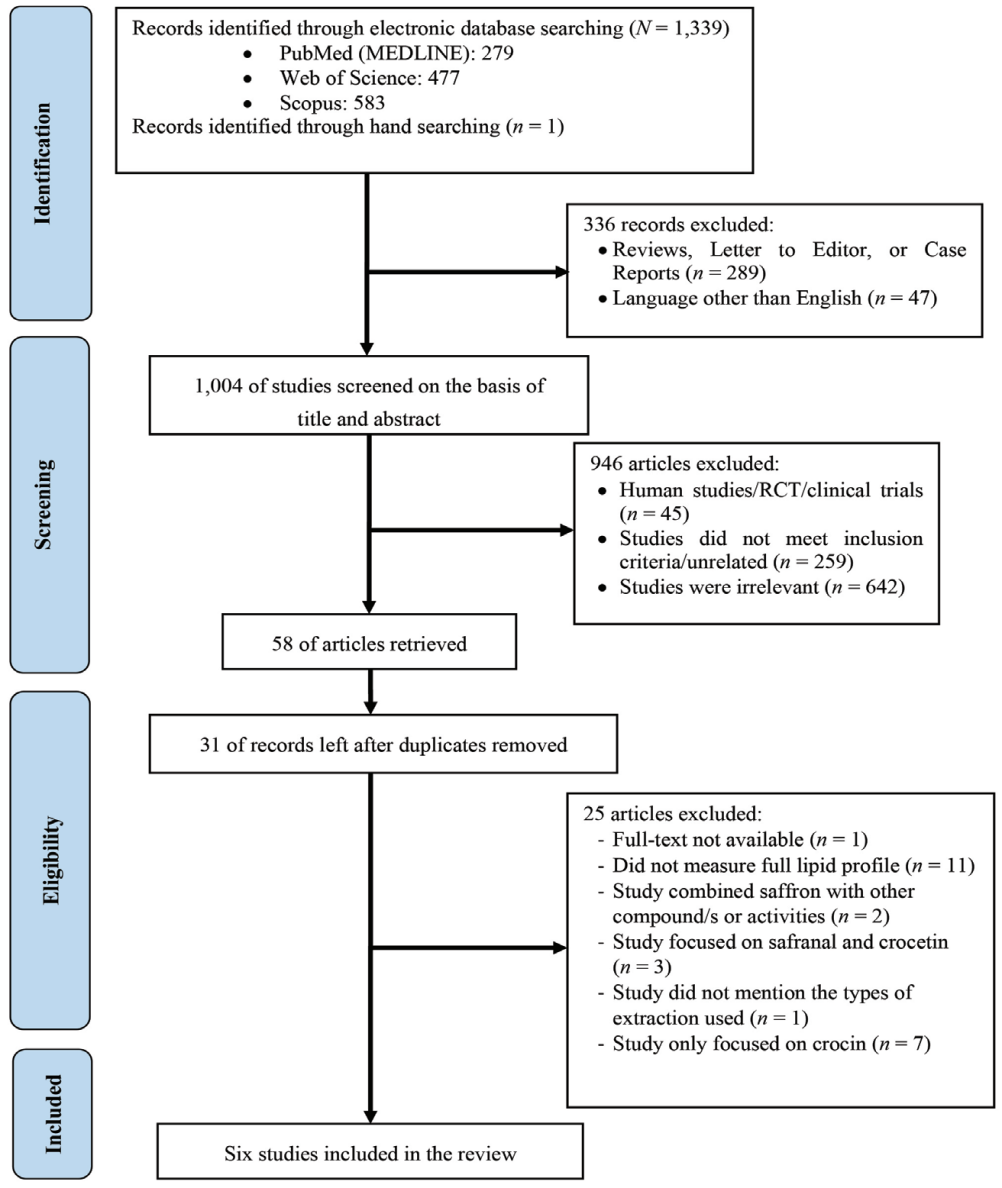
The PRISMA flow chart

There were two journals published in the year 2014, two journals published in 2016 and two journals published in 2017. A flow diagram of the literature selection including reasons for exclusion is shown in Figure 1.

### Study Characteristics

Of the six included studies, two studies (10–11) used Sprague-Dawley rats, three studies (12–14) used Wistar rats and only one study (15) used hamsters as their sample. All studies used male rodents as their subjects. The weight of the rats ranged between 200 g and 300 g, while the weight of the hamsters ranged between 80 g and 100 g. A summary of the characteristics of the studies is presented in Table 1.

Four studies were conducted based on an experimental design that compared the effect of SE-treated groups with control or placebo groups (12–15), while the other two studies included a positive control, either metformin (10) or orlistat (11). The studies used aqueous (*n* = 4), ethanolic (*n* = 1) and hydroalcoholic (*n* = 1) extracts.

There were four studies conducted in Iran (12–15) and two studies conducted in Malaysia (10–11). The animals were either given streptozotocin to induce diabetes or a high-fat diet (HFD) to induce hyperlipidaemia and obesity. There were five doses of SE used in these studies, ranging from 10 mg/kg/day to 100 mg/kg/day, with a duration ranging from 10 days to 8 weeks. SE was administered to rodents by four different routes: i) oral gavage (*n* = 2); ii) intraperitoneal injection (*n* = 2); iii) intra-gastric (*n* = 1) and iv) by mixing the SE with an HFD (*n* = 1).

The lipid profile of the rodents was evaluated by measuring the TC, TG, LDL-c and HDL-c levels in blood samples obtained at the end of the study. The sera were then analysed for biochemical parameters using standard commercial kits, an assay or an automatic analyser.

### The effects of saffron extracts on the lipid profile of animals

A total of six studies were included to evaluate the effects of SE on the lipid profile of diabetic, hyperlipidaemic and obese animals.

Samarghandian et al. (12) concluded that the administration of 10 mg/kg/day of SE for 4 weeks resulted in no significant differences between the treated and untreated groups, while 20 mg/kg/day for 4 weeks markedly decreased levels of TG (28.2%) and TC (20%) (*P* < 0.05). Another study (13) using a dose of 20 mg/kg/day with a duration of 4 weeks showed an increase in HDL (4.2%) and a decrease in TG (6%), TC (4.3%) and LDL (5.7%) levels between the treated and untreated groups, although the results were not significant.

There were five studies that used a dose of 40 mg/kg/day (10–14). However, only four studies showed significant results (10, 12–14). One of the studies (10) reported that, following the 6-week intervention, TC (30.8%), TG (29.1%) and LDL (42.9%) levels decreased in treated rats compared to untreated rats (*P* < 0.01), but no significant results were observed for HDL (*P* > 0.05). The percentage increase of HDL after saffron treatment was 9.2%. The results of another study (13) indicated that aqueous SE at a dose of 40 mg/kg/day for 4 weeks significantly reduced TC, TG and LDL levels (17.1%, 30% and 20.8%, respectively) (*P* < 0.05). Meanwhile, HDL increased by 37.5%. However, the HDL result was not statistically significant. The third study, conducted by the same author in different years (12), concluded that at dose 40 mg/kg/day with 4 weeks intervention period of aqueous SE, TG decreased significantly with a percentage decrease of approximately 38.2% (*P* < 0.01), LDL (38.7%) and TC decreased markedly with a percentage difference of approximately 65% from the untreated group (*P* < 0.05), while HDL level increased significantly with percentage up to 65% (*P* < 0.05). The fourth study (14) reported that aqueous SE at a dose of 40 mg/kg/day for 3 weeks reduced TC (30.7%), TG (39.2%) and LDL (45.3%) levels and increased HDL up to 19.5% (*P* < 0.05).

At a dose of 80 mg/kg/day, a study (14) concluded that SE markedly decreased TC (35.4%), TG (44.8%) and LDL (50%) levels in obese rats and significantly increased the serum HDL level up to 36.6% (*P* < 0.05) in 3 weeks. Another study (11) using 80 mg/kg/day concluded that in 8 weeks, SE markedly decreased the level of serum TC (28.5%) compared to the untreated group (*P* < 0.05). The results of the other parameters were not significant. Samarghandian et al. (13) stated that SE significantly decreased TG, TC and LDL levels at a dose of 80 mg/kg with a percentage decrease of 45%, 21.4% and 38.7% (*P* < 0.01), respectively, and increased HDL up to 56.3% based on a 4-week intervention (*P* < 0.05).

Only one study (15) administered 100 mg/kg/day of SE for 10 days. In this study, the percentage reduction of TG is 24.8%, TC (21.3%) and LDL (26.1%), while HDL increased up to 9.4%. However, the administration of SE in the treatment group did not significantly affect serum lipid profile compared to the untreated group (*P* > 0.05).

Of all the studies in our review, the minimum dose that showed significant results was 20 mg/kg/day, while the minimum duration that showed significant results was 3 weeks (Table 2).

## Discussion

In this systematic review, we evaluated the effect of SE on lipid profile in hyperlipidaemic experimental animal models. Lipid profile is one of the most important factors that can be changed in many diseases and are used to evaluate the conditions of the patients or subjects (16).

All six studies (10–15) in the review concluded that SE has a positive effect on the treatment of hyperlipidaemia, obesity, diabetes and its complications, although some of the results were not significant. However, none of the studies found that SE either worsened the condition or had negative effects. These positive results of SE on lipid profile could be due to the bioactive compounds contained in saffron, such as crocin and crocetin.

Crocin, one of the bioactive compounds of saffron, exerts its hypolipidaemic effect by inhibiting pancreatic lipase, leading to the malabsorption of fat and cholesterol (17). Further, crocin could elevate HDL levels by inhibiting cholesteryl ester transfer protein (CETP) (18), which is a plasma glycoprotein that reduces the concentration of HDL by transferring cholesteryl esters from HDL toward apo B-containing lipoproteins in exchange for TGs (19).

In addition, due to the presence of crocetin in saffron, it indirectly helps to reduce cholesterol levels, as crocetin has an inhibitory effect on TG (20) and LDL oxidation (21), thus contributing to the attenuation of atherosclerosis.

As anticipated, SE showed a greater ability than its single bioactive compound, crocin, to suppress appetite and dietary intake in patients with coronary artery disease (22). A previous in vivo study found that SE and crocin significantly decreased TG and TC levels in a dose-dependent manner (11). However, crocin showed higher anti-obesity effects, while SE had a more significant effect on reducing the appetite and food consumption of rats (11). This may be due to the synergistic effect of other bioactive compounds in SE, such as safranal, crocetin and picrocrocin.

Previously, in vitro studies have demonstrated that saffron and its bioactive compound, crocin, could reduce protein and gene expression of e-selectin, monocyte-endothelial cell interaction and endothelial activation (23–25).

Indirectly, SE has been proven to reduce lipid metabolism by reducing food consumption and decreasing appetite in obese rats due to the effect of safranal, one of the bioactive compounds of saffron (11). Another study confirmed that saffron exhibits an anorexigenic effect, leading to a reduction in body weight and blood leptin levels in Wistar rats (26). Lower food intake will indirectly reduce lipid levels in the blood, thus reducing the risk of atherosclerosis development.

In fact, SE could be used to prevent hyperlipidaemia. An in vivo study (10) reported that normal rats in a non-diabetic group (control) showed a decrease in LDL of about 21.8% after consumption of saffron hydroalcoholic extract at a dose of 40 mg/kg/day for 6 weeks.

The doses, experiment durations and types of SE play a significant role in determining positive and significant results. A study by Dehghan et al. (10) showed that hydroalcoholic SE at a dose of 40 mg/kg/day for 6 weeks significantly reduced TC (30.8%), TG (29.1%) and LDL (42.9%) levels, whereas HDL levels increased (9.2%). However, the HDL results were not significant. This was partially due to the different doses of saffron, as 40 mg/kg/day was not sufficient to affect HDL levels (10) compared to the doses used in previous research (27), which were 50 mg/kg/day and 100 mg/kg/day.

However, in comparison with the study by Samarghandian et al. (12), who used aqueous SE with the same dose (40 mg/kg/day) for 4 weeks, the results showed significant decreases in TC (23.5%), TG (38.2%) and LDL (38.7%) levels and a significant increase in HDL (65%). Therefore, the differences in the results could be due to another reason, possibly related to the use of different types of solvents in the process of saffron extraction. It has been reported that the type of solvent, time and method of extraction not only affect the diffusion rate of the components across the cell wall but also their stability (28). For instance, crocin, one of the main bioactive compounds of saffron, undergoes degradation during prolonged extraction in aqueous media with high water activity (28).

The preparation of an extract has a crucial impact on the accuracy of the results (29). A study (30) concluded that the selection of the most appropriate solvent for extracting the compounds of interest from a sample is an essential step in developing any extraction method. To extract the highest amount of active saffron components, the best solvent is methanol (50% v/v) followed by ethanol (50% v/v) and water (31). It has also been demonstrated that the use of alcohol or water-alcohol results in a higher extraction rate compared to water (28). Based on these studies, hydroalcoholic extracts should give better results than aqueous SE. However, a study indicated that approximately 50% of crocetin esters and 70% of picrocrocin from an aqueous SE were bio-accessible under in vitro gastrointestinal digestion conditions (32). However, evidence regarding the bioaccessibility of hydroalcoholic SE is not widely available.

A study by Samarghandian et al. (13) that used a dose of 40 mg/kg/day for 4 weeks showed a significant decrease in TG (30%), LDL (20.8%) and TC (17.1%) levels and an increase of HDL (37.5%), although the HDL result was not significant. However, in 2017, Samarghandian et al. (12) conducted a study using the same dose (40 mg/kg/day), duration (4 weeks) and type of extraction, and HDL increased significantly (65%). This may have been due to improvements made by the researchers compared to the previous studies. In 2014, they used 180 g–220 g Wistar rats (13). According to the growth and length chart (33) for Wistar rats, that weight was equivalent to about 6 weeks old–8 weeks old of age. In 2017, adult Wistar rats (weight 250 g–330 g) were used, equivalent to about 8 weeks old–12 weeks old of age. The age of the animals used is an important factor when conducting an experiment. Inconsistent choice of age in rodent models has the potential to impact data quality, potentially increasing variability and reducing their relevance to the human disease being studied (34). The most commonly used age for animal models is 8 weeks old–12 weeks old, which is due to the ongoing developmental processes and changes in physiology, which may have a large impact on experimental variables (34). The 6-week-old–8-week-old Wistar rats used in the study may have affected the results indirectly and caused the study to produce less significant results.

Of the studies that used SE at a dose of 80 mg/kg/day (11, 13–14), two showed positive results (13–14), with a significant decrease in TC (up to 35.4%), TG (45%) and LDL (up to 50%) levels and a significant increase in HDL (56.3%) levels with durations of 3 weeks and 4 weeks, respectively. However, one of the studies (11) only showed a significant decrease in TC levels (28.5%), while the other results (TG, LDL and HDL) were not significant. The inconsistency in the results could be due to the different methods of administration of saffron, as the researchers mixed the SE with HFD (11). This may have caused the rats to ingest an inconsistent or inaccurate amount of the extracts. Gavage (oesophageal or gastric) is often used in research settings, instead of mixing substances in water or food, to ensure precise and accurate dosing of animals (35). Hoshyar et al. (14) used an oral gavage method, while Samarghandian et al. (13) injected the SE intraperitoneally. These methods are in line with those used in other studies, indicating that both of these methods could yield significant results (27, 36).

One of the common routes of administration used in rodent studies is oral gavage (37). This is because of the higher accuracy of the dosages, reliable timing and faster absorption of unstable or unpalatable compounds compared to dietary administration (38). However, if the technique is not done properly, there is a risk of complications, such as inadvertent tracheal administration, aspiration pneumonia, oesophageal perforation, oesophageal impaction or gastric rupture, which could increase the morbidity and mortality of the rodents (39). Hoggatt et al. (37) suggested that the needles should be precoated with sucrose to decrease the signs of stress and improve animal welfare during oral gavage.

Another frequent route of administration is through intraperitoneal injection. The advantage of this method is rapid absorption of the substance due to the large surface area and abundant blood supply of the abdominal cavity (38). However, this method is not suitable for irritating substance because it can cause peritonitis (40). In addition, repeated injections may cause tissue reactions and adhesions in long-term studies (38). There is also a risk of puncturing other abdominal organs, such as the intestine or kidney, if it is not done properly.

Another method is to mix the substance with food or drinking water. This is the simplest method of administration, as no handling or restraining of animals is required. Thus, this method will not cause stress to the animals and the digestion is able to occur under normal physiological conditions. However, this method is not suitable when dealing with substances that are distasteful, not soluble in water or which could cause irritation to the gastrointestinal mucosa (38). Moreover, the mixing of substances into the food must be done carefully to avoid food decomposition. The animals might refuse to eat and drink, and this could cause a loss of weight and dehydration. It is also important to record the food and water intake of the rats daily before the intervention procedure so that the quantity of the substance to be mixed with the food or drinking water can be properly estimated (38). Moreover, rats must be caged individually to enable accurate measurement of food or water intake. However, this living environment may create stress in the rats, thus reducing the effectiveness of this administration method (38).

A study (15) administered 100 mg/kg/day of SE for 10 days, and the results showed no significant difference between the treated and untreated groups. This could have been due to the relatively small sample size (*n* = 26). The power of a study increases as the sample size increases and the ideal minimum power of a study is 80% (41). Additionally, the sample size calculation directly influences the research findings, as very small samples could undermine the internal and external validity of a study and result in no significant findings (42).

There were two studies (10–11) that included a positive control (metformin and orlistat, respectively) for comparison. TC and TG levels decreased significantly with the administration of metformin and ethanolic SE. However, metformin reduced TC (5.4%) and TG (11.7%) levels more than SE. Hence, both SE and metformin were effective in combatting hyperlipidaemia. Another study by Mashmoul et al. (11) showed that both SE and orlistat could decrease TG, TC and LDL levels and increase HDL, although some of the results were not significant. However, orlistat has been reported to have several gastrointestinal side effects, including oily spotting, frequent loose stools and flatulence (43).

Regarding saffron toxicity, some in vivo studies showed an increase in blood urea nitrogen (BUN) and creatinine levels, which indicate kidney dysfunction, and a significant decrease in red blood cells, white blood cells and haemoglobin levels (44). An increase in liver enzymes, alanine aminotransferase (ALT) and aspartate aminotransferase (AST) could also be seen in sub-chronic saffron toxicity (45). These signs were not observed in all the studies included in this review and Dehghan et al. (10) confirmed that saffron is highly biocompatible for clinical applications, with no signs of toxicity at the tested dose and no recorded mortality in their experiment. Regarding the lethal dose (LD_50_) value of aqueous SE, Hosseinzadeh et al. (46) reported a value of 4,120 ± 556 mg/kg for oral exposure in BALB/c mice, and for intraperitoneal administration the LD_50_ value of saffron stigma was 1.6 g/kg in mice. We can thus conclude that, for future studies, SE is safe below the LD_50_ values stated.

The results of this review validate the potential of SE for treating one of the risk factors of atherosclerosis—hyperlipidaemia—as well as preventing related complications. Of all the in vivo studies in our review, the minimum SE dose that showed significant results was 20 mg/kg/day, while the minimum duration that showed significant results was 3 weeks.

These findings are in contrast the results of a meta-analysis of randomised clinical trials (RCTs) done on patients with metabolic disorders by Roshanravan et al. (47). In their meta-analysis, the authors concluded that the effects of saffron on the lipid profile of patients was only significant after 6 weeks–12 weeks of treatment, while crocin supplementation of 5 mg/d, 15 mg/d and 30 mg/d did not show any significant effects on lipid profile with the same treatment duration. The difference in the effects of saffron and crocin is related to the patients’ stage of disease (47).

The results regarding the minimum dose and duration in our study differ because the subjects and types of studies were different. While Roshanravan et al. (47) conducted a systematic review and meta-analysis on RCTs, our systematic review focused on in vivo studies. Therefore, to achieve the maximum benefit of SE, more clinical trials with different doses and durations are needed. In addition, other in vivo studies could use the articles gathered in this systematic review to estimate the minimum dose, duration of treatment, type of animals used and minimum sample size required to conduct their experiments and research.

This study was in alignment with the WHO Global Action Plan for the prevention and control of non-communicable diseases 2013–2020, which aims to promote and support the national capacity for high-quality research and development to help prevent and control non-communicable diseases (48).

This systematic review has several strengths. To the best of our knowledge, this is the first study to review the effects of SE on lipid profile in in vivo studies. A broad search term was used to capture the largest possible number of publications on this topic. There were two independent reviewers during the article selection and data extraction to reduce bias. A third independent person was included if there were any disagreements in the selection and extraction. However, the results of this study may have limited generalisability due to selection bias.

Although the results of our review are encouraging, with most of the studies displaying positive effects of saffron on health conditions related to hyperlipidaemia, limitations still exist in terms of confirming the therapeutic efficacy of SE. For example, potential biases existed because Iranian research groups conducted most of the in vivo experiments. Iran is known to be one of the greatest producers of saffron, and nearly 90% of saffron is produced in Iran (49). Thus, most of the research regarding saffron comes from Iran. Institutions in other countries are encouraged to validate Iranian research, as saffron appears to be a promising medicinal plant, particularly as a hypolipidaemic agent. Further, this review was limited to only three electronic databases (MEDLINE, Web of Science and Scopus), to accessible full-text articles and to articles published in English. However, these databases provide wide coverage of published medical journals. In fact, most high-quality and high-impact journals are published in English. Furthermore, due to the limited intervention durations in the included studies, we could not determine the long-term effects of SE on lipid profile. The included studies also used different types of extractions, doses and routes of administration. Therefore, the results varied and it is difficult to come to a final conclusion.

## Conclusion

This systematic review on six studies demonstrated that SE exhibits hypolipidaemic and anti-hyperlipidaemic effects. In fact, SE is nearly as effective in reducing cholesterol levels as standard therapies, such as orlistat and metformin. Further studies should be done to define the minimum effective dose of SE, the minimum duration of intervention and the best SE preparation method to achieve the maximum anti-hyperlipidaemic or hypolipidaemic effects of saffron.

## Figures and Tables

**Table 1:** Summary table on the effect of saffron extract on lipid profile in hyperlipidaemic experimental animal model

No.	Author (year)/Country	Aim of study	Test animal	Types of extract used	Dose, duration and route	Hypolipidaemic effects of saffron	Conclusion
1.	Dehghan et al. (2016)/Malaysia (10)	To establish the effectiveness of saffron on diabetic parameters in vitro and combined with resistance exercise intervention in vivo models	Sprague-Dawley rats - Sex: Male - Weight: 250 g ± 15 g - Age: 8 weeks old - Sample size: *n* = 70	Hydro-alcoholic extract	40 mg/kg/day for 6 weeks intra-gastrically	- TC, TG, and LDL levels decreased in treated rats compared to untreated (*P* < 0.01) - No significant differences were observed in the HDL (*P* > 0.05)	The consumption of the herbal plant saffron combined with resistance exercise is a strong therapeutic effective factor on diabetic parameters in vivo
2.	Hoshyar et al. (2016)/Iran (14)	To evaluate and compare the effects of saffron stigma, petal, and their mixture on lipid profile, liver enzymes, adipose-derived hormones, and on the risk of atherosclerosis and insulin resistance in obese rats in order to clarify the cellular mechanism behind the anti-obesity properties of saffron	Wistar albino rats - Sex: Male - Weight: 200 g ± 10 g - Age: 60 days old - Sample size: *n* = 56	Aqueous extract	40 mg/kg/day and 80 mg/kg/day for 3 weeks by oral gavage	- SE (40 mg/kg and 80 mg/kg) markedly decreased the serum TC, TG and LDL in obese rats and increased the serum HDL (*P* < 0.05)	SE particularly the mixture of extracts from stigma and petal, ameliorated dyslipidaemia in obese rats, leading to decreased atherosclerosis and insulin resistance
3.	Mashmoul et al. (2014)/Malaysia (11)	To evaluate anti-obesity effect of ethanolic extract of saffron and its pure bioactive compound, crocin in a model of HFD-induced obesity	Sprague-Dawley rats - Weight: 217 g ± 25 g - Sample size: *n* = 42	Ethanolic extract of saffron	40 mg/kg and 80 mg/kg for 8 weeks. Fed to rats by mixing with HFD	- SE markedly decreased the serum TC at 80 mg/kg dose compared to untreated (*P* < 0.05)	The potential of ethanolic extract of saffron and crocin in reducing obesity and aiding in weight management was confirmed in this study
4.	Samarghandian et al. (2017)/Iran (12)	To evaluate the effects of saffron on the plasma levels of glucose, lipids, oxidant and antioxidant balance, and the changes of pro-inflammatory genes expression in the abdominal aorta to verify the protective effects of saffron on vascular complication of diabetes mellitus (DM) in rats	Wistar rats - Sex: Male - Weight: 250 g–300 g - Sample size: *n* = 45	Aqueous extract of saffron	10 mg/kg/day, 20 mg/kg/day and 40 mg/kg/day for 4 weeks through intraperitoneal injection	- Saffron significantly decreased TG and TC at 20 mg/kg (*P* < 0.05) - Saffron significantly decreased TG at 40 mg/kg (*P* < 0.01) - Saffron markedly decreased LDL-c and TC at dose 40 mg/kg (*P* < 0.05), while increase HDL-c at dose 40 mg/kg (*P* < 0.05)	Saffron could be used as a treatment against DM and its vascular complications
5.	Samarghandian et al. (2014)/Iran (13)	To evaluate the effect of SE on a model of diabetes mellitus and its effects on serum lipid profiles, tumour necrosis factor-alpha (TNF-*α*), a marker for inflammation, and oxidative stress parameters in this process	Wistar albino rats - Sex: Male - Weight: 180 g–220 g - Sample size: *n* = 50	Aqueous extract of saffron	20 mg/kg/day, 40 mg/kg/day and 80 mg/kg/day for 4 weeks through intra-peritoneal injection	- Saffron significantly decreased TG, TC and LDL at dose 80 mg/kg (*P* < 0.01) and increase HDL at dose of 80 mg/kg (*P* < 0.05)	SE may reduce hyperglycaemia and hyperlipidaemia risk and reduce the oxidative stress in diabetic encephalopathy rats
6.	Vakili et al. (2017)/Iran (15)	To investigate the effects of saffron consumption on lipid profiles, especially the reduction of blood cholesterol, and lipid peroxidation in male hamsters under HFD	Hamster - Sex: Male - Weights: 80 g–100 g - Sample size: *n* = 26	Aqueous extract of saffron	100 mg/kg/day saffron for 10 days through oral gavage	- Administration of SE in the HFD and saffron group did not significantly affect the serum lipid profile compared to the HFD group (*P* > 0.05)	SE can be considered as one of the candidates of phytomedicine for prevention and treatment of cardiovascular diseases

**Table 2:** Minimum dose and duration that showed significant results on the lipid profile

Author (year) (References)	Types of SE	Minimum dose and duration that showed significant results	Lipid profile	Increase and decrease (%)	*P*-value
Samarghandian et al. (2017) (12)	Aqueous extract	Minimum dose: 20 mg/kg/day (4 weeks)	TG	Reduced by 28.2	< 0.05
			TC	Reduced by 20.0	
Hoshyar et al. (2016) (14)	Aqueous extract	Minimum duration: 3 weeks (40 mg/kg/day)	TG	Reduced by 39.2	< 0.05
			TC	Reduced by 30.7	
			HDL	Increased by 19.5	
			LDL	Reduced by 45.3	

## References

[1] Karr S (2017). Epidemiology and management of hyperlipidemia. Am J Manag Care.

[2] Gaziano TA, Bitton A, Anand S, Abrahams-Gessel S, Murphy A (2010). Growing epidemic of coronary heart disease in low- and middle-income countries. Curr Probl Cardiol.

[3] Nelson RH (2013). Hyperlipidemia as a risk factor for cardiovascular disease. Prim Care.

[4] World Health Organization (WHO) (2008). Global Health Observatory (GHO) data. Raised cholesterol: situation and trends. WHO [Internet].

[5] Zhao LY, Huang W, Yuan QX, Cheng J, Huang ZC, Ouyang LJ (2012). Hypolipidaemic effects and mechanisms of the main component of *Opuntia dillenii* Haw. polysaccharides in high-fat emulsion-induced hyperlipidaemic rats. Food Chem.

[6] Hausenblas HA, Heekin K, Mutchie HL, Anton S (2015). A systematic review of randomized controlled trials examining the effectiveness of saffron (*Crocus sativus* L.) on psychological and behavioral outcomes. J Integr Med.

[7] Ghaffari S, Roshanravan N (2019). Saffron: an updated review on biological properties with special focus on cardiovascular effects. Biomed Pharmacother.

[8] Kamalipour M, Akhondzadeh S (2011). Cardiovascular effects of saffron: an evidence-based review. J Tehran Heart Cent.

[9] Moher D, Liberati A, Tetzlaff J, Altman DG, PRISMA Group (2009). Preferred reporting items for systematic reviews and meta-analyses: the PRISMA statement. PLoS Med.

[10] Dehghan F, Hajiaghaalipour F, Yusof A, Muniandy S, Hosseini SA, Heydari S (2016). Saffron with resistance exercise improves diabetic parameters through the GLUT4/AMPK pathway *in-vitro* and *in-vivo*. Sci Rep.

[11] Mashmoul M, Azlan A, Yusof BNM, Khaza’ai H, Mohtarrudin N, Boroushaki MT (2014). Effects of saffron extract and crocin on anthropometrical, nutritional and lipid profile parameters of rats fed a high fat diet. J Funct Foods.

[12] Samarghandian S, Azimi-Nezhad M, Farkhondeh T (2017). Immunomodulatory and antioxidant effects of saffron aqueous extract (*Crocus sativus* L.) on streptozotocin-induced diabetes in rats. Indian Heart J.

[13] Samarghandian S, Azimi-Nezhad M, Samini F (2014). Ameliorative effect of saffron aqueous extract on hyperglycemia, hyperlipidemia, and oxidative stress on diabetic encephalopathy in streptozotocin induced experimental diabetes mellitus. Biomed Res Int.

[14] Hoshyar R, Amini Z, Valavi M, Zare Beyki M, Mehrpour O, Hosseinian M (2016). Anti-dyslipidemic properties of saffron: reduction in the associated risks of atherosclerosis and insulin resistance. Iranian Red Crescent Med J.

[15] Vakili S, Savardashtaki A, Momeni Moghaddam MA, Nowrouzi P, Khabbaz Shirazi M, Ebrahimi G (2017). The effects of saffron consumption on lipid profile, liver enzymes, and oxidative stress in male hamsters with high fat diet. Trends Pharm Sci.

[16] Mohtashami A, Entezari MH (2016). Effects of *Nigella sativa* supplementation on blood parameters and anthropometric indices in adults: a systematic review on clinical trials. J Res Med Sci.

[17] Sheng L, Qian Z, Zheng S, Xi L (2006). Mechanism of hypolipidemic effect of crocin in rats: crocin inhibits pancreatic lipase. Eur J Pharmacol.

[18] Javandoost A, Afshari A, Nikbakht-Jam I, Khademi M, Eslami S, Nosrati M (2017). Effect of crocin, a carotenoid from saffron, on plasma cholesteryl ester transfer protein and lipid profile in subjects with metabolic syndrome: a double blind randomized clinical trial. ARYA Atheroscler.

[19] Chyou JY, Mega JL, Sabatine MS, Antman EM, Sabatine MS (2013). Chapter 4-Pharmacogenetics. Cardiovascular therapeutics: a companion to Braunwald’s heart disease.

[20] Lee IA, Lee JH, Baek NI, Kim DH (2005). Antihyperlipidemic effect of crocin isolated from the fructus of *Gardenia jasminoides* and its metabolite Crocetin. Biol Pharm Bull.

[21] Zheng S, Qian Z, Sheng L, Wen N (2006). Crocetin attenuates atherosclerosis in hyperlipidemic rabbits through inhibition of LDL oxidation. J Cardiovasc Pharmacol.

[22] Abedimanesh N, Bathaie SZ, Abedimanesh S, Motlagh B, Separham A, Ostadrahimi A (2017). Saffron and crocin improved appetite, dietary intakes and body composition in patients with coronary artery disease. J Cardiovasc Thorac Res.

[23] Alicezah MK, Rahman T, Froemming GR, Ahmad R, Nawawi H (2014). Saffron and its active compound, crocin inhibits endothelial activation in stimulated human coronary artery endothelial cells. Atherosclerosis.

[24] Alicezah M, Ahmad R, Rahman T, Froemming GR, Nawawi H (2015). The effect of saffron and its bioactive compound; crocin against monocyte-endothelial cell interaction in human coronary arterial endothelial cells. Atherosclerosis.

[25] Kasim NM, Abd Rahim IN, Bakar NA, Ahmad R, Rahman TA, Froemming GR (2020). Saffron and its active compound, crocin inhibit E-selectin in LPS-stimulated human coronary artery endothelial cells. Atherosclerosis.

[26] Kianbakht S, Hashem Dabaghian F (2015). Anti-obesity and anorectic effects of saffron and its constituent crocin in obese Wistar rat. J Med Plants.

[27] Asdaq SM, Inamdar MN (2010). Potential of *Crocus sativus* (saffron) and its constituent, crocin, as hypolipidemic and antioxidant in rats. Appl Biochem Biotechnol.

[28] Orfanou O, Tsimidou M (1996). Evaluation of the colouring strength of saffron spice by UV-vis spectrometry. Food Chem.

[29] Heydari S, Haghayegh GH (2014). Extraction and microextraction techniques for the determination of compounds from saffron. Can Chem Trans.

[30] Gazerani S, Sani A, Tajalli F (2013). Effect of solvent extraction on qualitative parameters of saffron edible extract. Res Rev Biosci.

[31] Koocheki A, Khajeh-Hosseini M (2019). Saffron: science, technology and health.

[32] Kyriakoudi A, Tsimidou MZ, O’Callaghan YC, Galvin K, O’Brien NM (2013). Changes in total and individual crocetin esters upon in vitro gastrointestinal digestion of saffron aqueous extracts. J Agric Food Chem.

[33] Xie T, Zaidi H (2013). Age-dependent small-animal internal radiation dosimetry. Mol Imaging.

[34] Jackson SJ, Andrews N, Ball D, Bellantuono I, Gray J, Hachoumi L (2017). Does age matter? The impact of rodent age on study outcomes. Lab Anim.

[35] Turner PV, Brabb T, Pekow C, Vasbinder MA (2011). Administration of substances to laboratory animals: routes of administration and factors to consider. J Am Assoc Lab Anim Sci.

[36] Mohajeri D, Mousavi G, Mesgari M, Doustar Y, Khayat Nouri MH (2007). Subacute toxicity of *Crocus sativus* L.(saffron) stigma ethanolic extract in rats. Am J Pharmacol Toxicol.

[37] Hoggatt AF, Hoggatt J, Honerlaw M, Pelus LM (2010). A spoonful of sugar helps the medicine go down: a novel technique to improve oral gavage in mice. J Am Assoc Lab Anim Sci.

[38] Nebendahl K, Krinkle GJ (2000). Routes of administration. The handbook of experimental animals: the laboratory rat.

[39] Germann PG, Ockert D (1994). Granulomatous inflammation of the oropharyngeal cavity as a possible cause for unexpected high mortality in a Fischer 344 rat carcinogenicity study. Lab Anim Sci.

[40] Gotloib L, Wajsbrot V, Shostak A (2005). A short review of experimental peritoneal sclerosis: from mice to men. Int J Artif Organs.

[41] Suresh K, Chandrashekara S (2012). Sample size estimation and power analysis for clinical research studies. J Hum Reprod Sci.

[42] Faber J, Fonseca LM (2014). How sample size influences research outcomes. Dental Press J Orthod.

[43] Guh DP, Zhang W, Bansback N, Amarsi Z, Birmingham CL, Anis AH (2009). The incidence of co-morbidities related to obesity and overweight: a systematic review and meta-analysis. BMC Public Health.

[44] Bostan HB, Mehri S, Hosseinzadeh H (2017). Toxicology effects of saffron and its constituents: a review. Iran J Basic Med Sci.

[45] Muosa F, AL-Rekabi K, Askar SJ, Yousif EH (2015). Evaluation of the toxic effect of ethanolic extract of saffron in male mice after subchronic exposure. Donnish J Pharm Pharmacol.

[46] Hosseinzadeh H, Sadeghi Shakib S, Khadem Sameni A, Taghiabadi E (2013). Acute and subacute toxicity of safranal, a constituent of saffron, in mice and rats. Iran J Pharm Res.

[47] Roshanravan B, Samarghandian S, Ashrafizadeh M, Amirabadizadeh A, Saeedi F, Farkhondeh T (2022). Metabolic impact of saffron and crocin: an updated systematic and meta-analysis of randomised clinical trials. Arch Physiol Biochem.

[48] World Health Organization (WHO) (2013). Global action plan for the prevention and control of noncommunicable diseases 2013–2020 [Internet].

[49] Moghaddasi MS (2010). Saffron chemicals and medicine usage. J Med Plants Res.

